# Supplementary material for: Functional genomics screens reveal a role for TBC1D24 and SV2B in antibody-dependent enhancement of dengue virus infection
Source: J Virol. 2024 Oct 8;98(11):e01582-24. doi: 10.1128/jvi.01582-24 (PMC11578089; doi:10.1128/jvi.01582-24)
Supplement: Supplemental figures — Figures S1 to S11. [file jvi.01582-24-s0001.pdf]

Supplemental Materials

Supplemental Table 1: Output of MAGeCK analysis of genome-wide screen.
Supplemental Table 2: Complete list of genes and guides used for targeted sub-library screen.
Supplemental Table 3: Output of MAGeCK analysis of targeted sub-library screen.
Supplemental Table 4: Sequences of sgRNA oligos and PCR primers.
Supplemental Table 5: Raw percentages of infected cells at each antibody concentration / serum dilution as enumerated by flow cytometry for data displayed in relevant main and supplemental figures

Figure S1

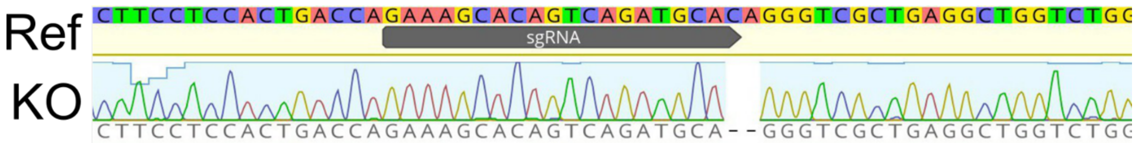

Fig S1: Genotyping of K562 FcgRIIa KO clone.
Sanger sequencing of locus targeted by sgRNA in the K562 FcgRIIa KO clonal line. Traces were aligned to WT reference sequence (“Ref”) to identify the indicated deletion.

Figure S2

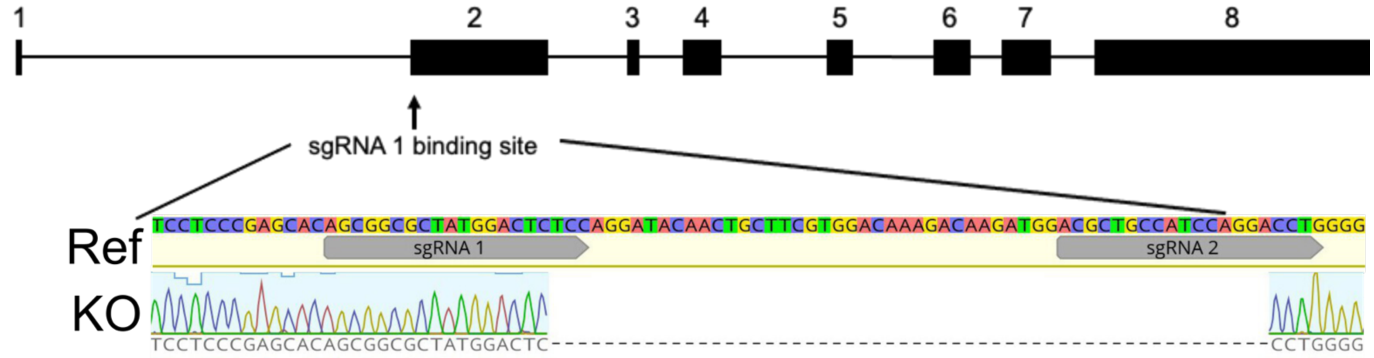

Fig S2: Genotyping of K562 TBC1D24 KO clone.
(Top) schematic of TBC1D24 exons (boxes) and introns (lines). (Bottom) Sanger sequencing of loci targeted by sgRNA1 (Table S4) in the K562 TBC1D24 KO clonal cell line. Traces were aligned to WT reference sequence ("Ref") to identify the indicated deletion within exon 2.

Figure S3

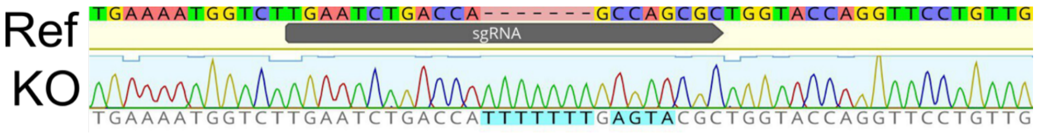

Fig S3: Genotyping of K562 SV2B KO clone.
Sanger sequencing of locus targeted by sgRNA in K562 SV2B KO clonal cell line. Traces were aligned to WT reference sequence ("Ref") to identify a 7 bp insertion and 4 bp missense mutation

Figure S4

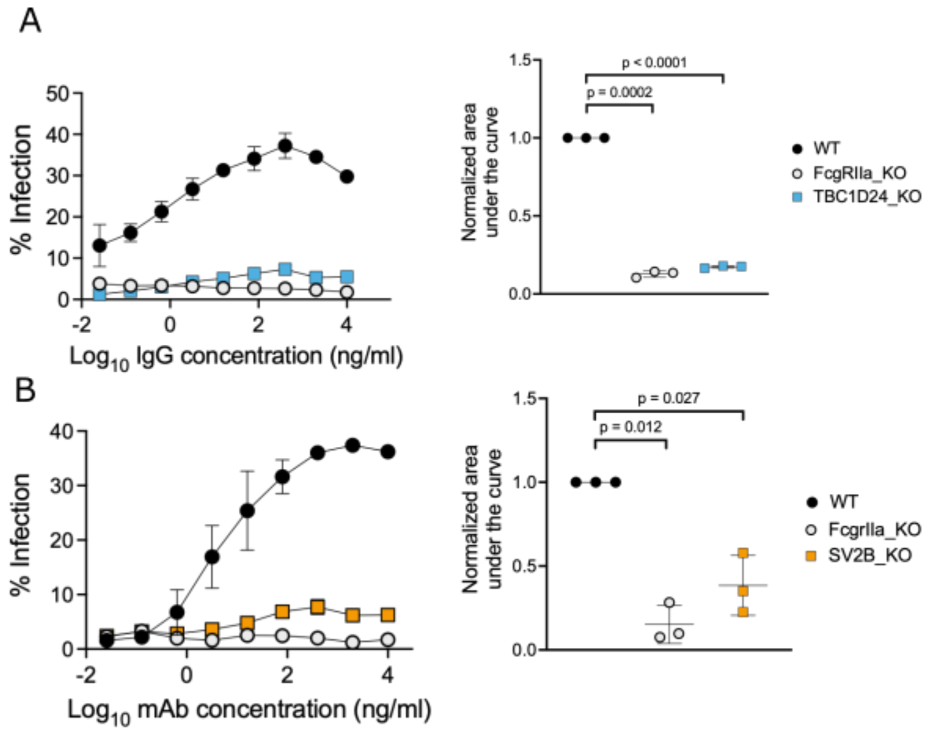

Fig S4: Functional validation of TBC1D24 and SV2B in ADE assays

A-B: (Left) The indicated K562 cells were infected via ADE using DENV2-GFP in the presence of serially diluted mouse anti-DENV IgG monoclonal antibody DV2-70 used in CRISPR screens. Data points represent the mean of three independent experiments normalized to the peak infection level of WT cells, and the error bars represent the standard deviation. (Right) Quantification of area under the curve normalized to WT K562 cells from three independent dose-response ADE experiments (data points), each performed in duplicate wells. Horizontal lines and error bars indicate mean and standard deviation, respectively. In each experiment, a FcgRIIa KO clone was included as a control. P-values shown are from multiple independent paired student's t-tests adjusted using the Benjamini-Hochberg method.

Figure S5

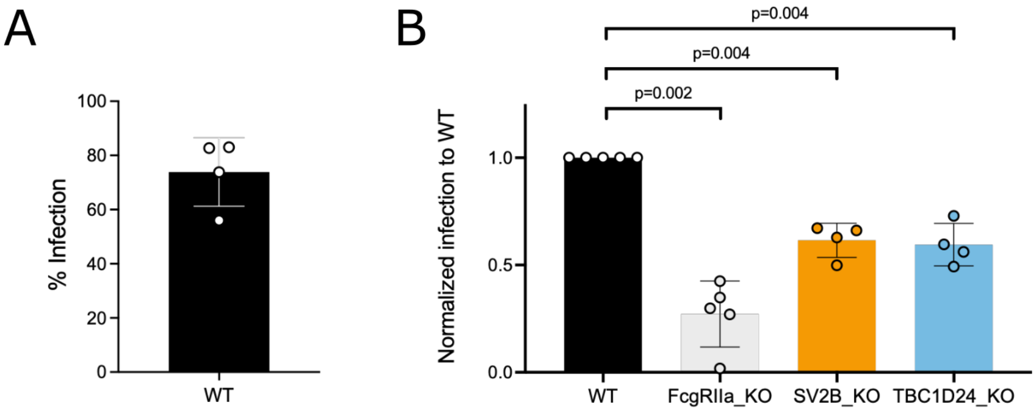

Fig S5: Levels of infection and functional validation of TBC1D24 and SV2B in high MOI ADE assays

The indicated K562 cells were infected via ADE using DENV2-GFP at an MOI of 24 in the presence of **(A)** mouse IgG2a DV2-70 (1.25 µg/mL) or **(B)** human IgG1 J9 (80 ng/ml) at a concentration that resulted in peak enhancement of infection as determined in dose-response assays. Bars represent the mean of at least four independent experiments depicted by data points; error bars represent standard deviation.

Figure S6

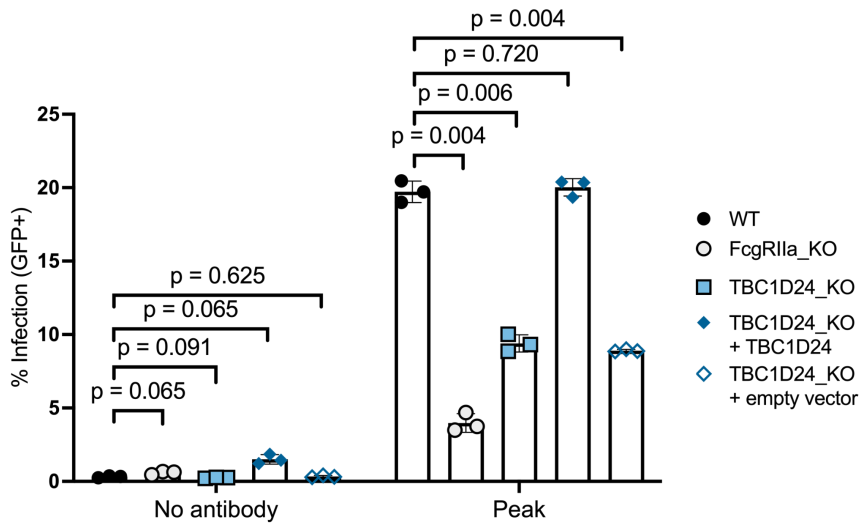

Fig S6: Comparison of DENV2 infection of K562 cells in the presence or absence of IgG
Efficiency of DENV2-GFP infection of the indicated K562 cells in the absence of antibody or in the presence of J9 antibody at a concentration that resulted in peak enhancement of infection as determined from dose-response assays in Figure 2. Bars represent the mean of three independent experiments depicted by data points and error bars represent standard deviation.

Figure S7

Clone	KO Score
KO_1	92
KO_2	nd
KO_3	97
KO_4	nd

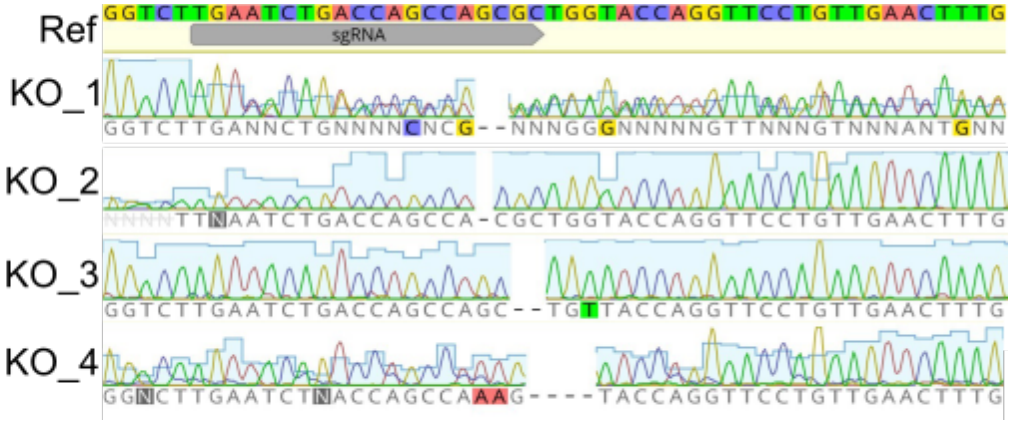

Fig S7: Genotyping of K562-DCSIGN SV2B KO clones.
(Right) Sanger sequencing of locus targeted by sgRNA in the K562-DCSIGN SV2B KO clones. Traces were aligned to WT reference sequence and heterogeneous mutations deconvoluted using Inference of CRISPR Edits (ICE; https://ice.synthego.com/#/). (Left) KO scores as determined by ICE; nd = not determined.

Figure S8

Clone	KO Score
KO_1	100
KO_2	92
KO_3	100
KO_4	93
KO_5	100
KO_6	100

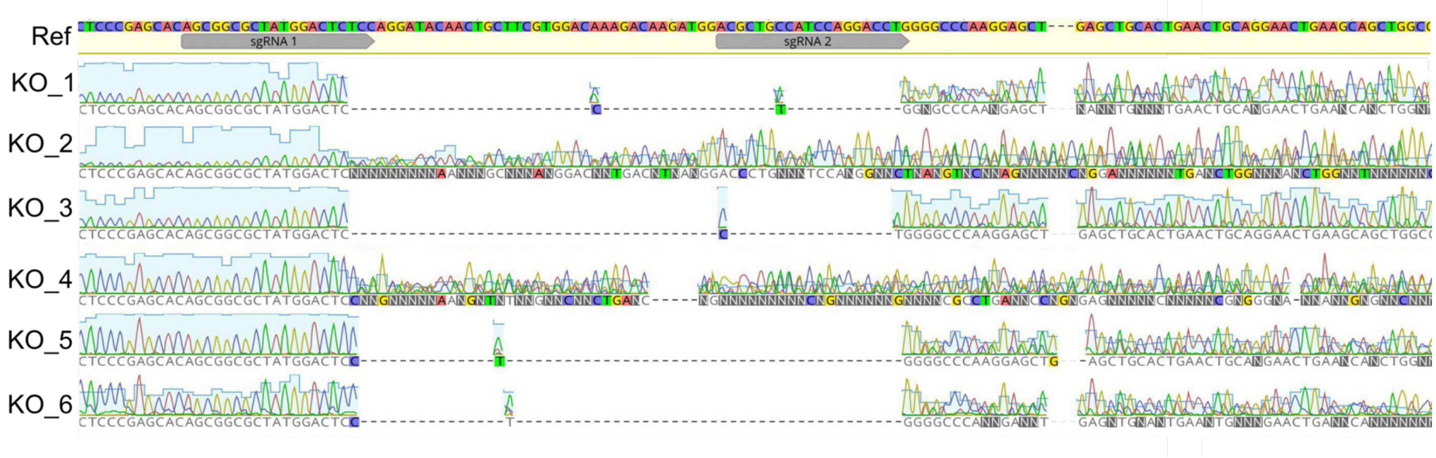

Fig S8: Genotyping of TBC1D24 KO K562-DCSIGN Cells.
(Right) Sanger sequencing of locus targeted by gRNA in K562-DCSIGN TBC1D24 KO clones. Traces were aligned to WT reference sequence and heterogeneous mutations deconvoluted using ICE (https://ice.synthego.com/#/). (Left) KO scores as determined by ICE.

Figure S9

Clone	KO Score
KO_1	100
KO_2	100
KO_3	100
KO_4	100
KO_5	95
KO_6	100
KO_7	100
KO_8	100
KO_9	96
KO_10	100
KO_11	100

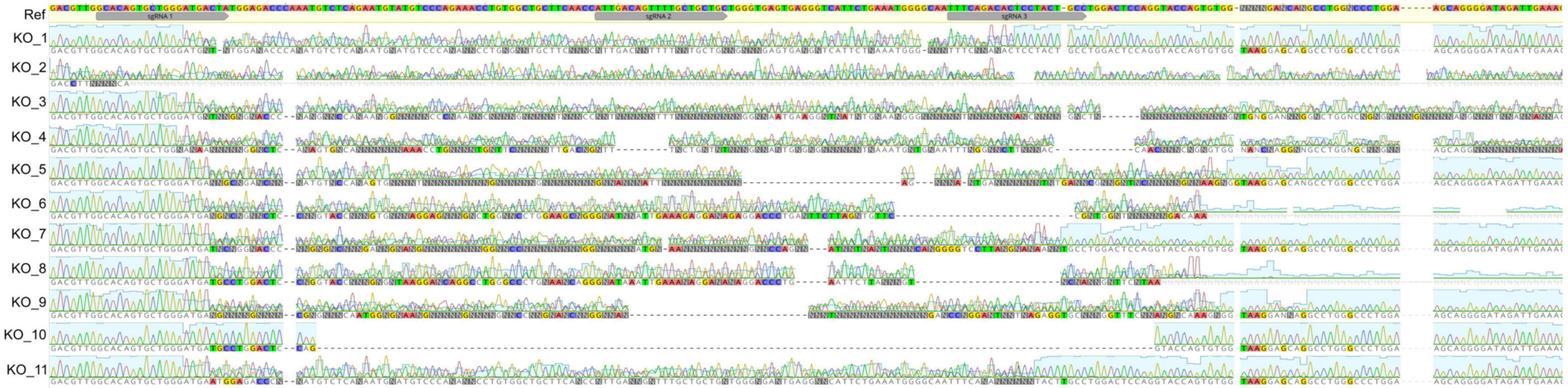

Fig S9: Genotyping of FcgRIIa KO K562-DCSIGN Cells.
(Right) Sanger sequencing of locus targeted by gRNA K562-DCSIGN FcgRIIA KO clones. Traces were aligned to WT reference sequence and heterogeneous mutations deconvoluted using ICE (https://ice.synthego.com/#/). (Left) KO scores as determined by ICE.

Figure S10

Clone	KO Score
KO_1	100
KO_2	100
KO_3	100
KO_4	100

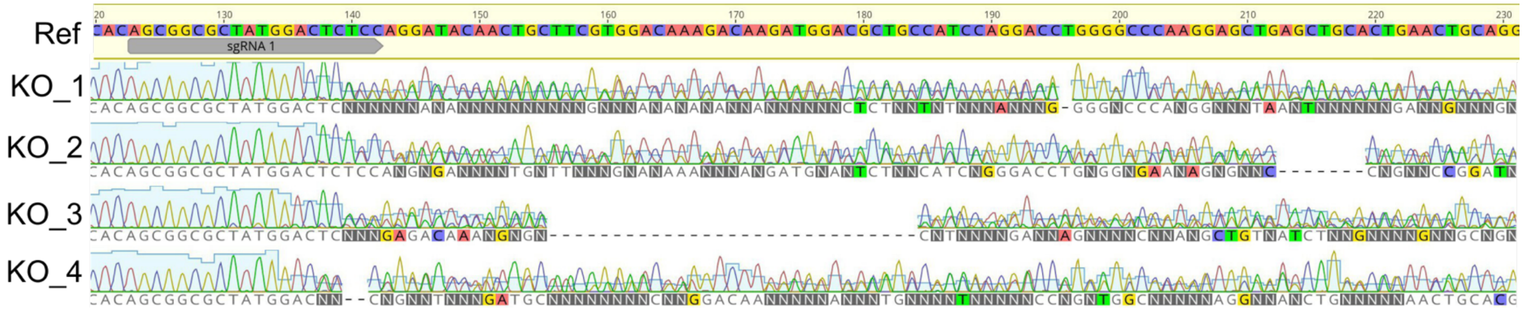

Fig S10: Genotyping of TBC1D24 KO U937 Cells.
Sanger sequencing of locus targeted by gRNA in U937 TBC1D24 KO clones. Traces were aligned to WT reference sequence and heterogeneous mutations deconvoluted using ICE (https://ice.synthego.com/#/). (Left) KO scores as determined by ICE.

Figure S11

Clone	KO Score
KO_1	100
KO_2	100
KO_3	100
KO_4	100

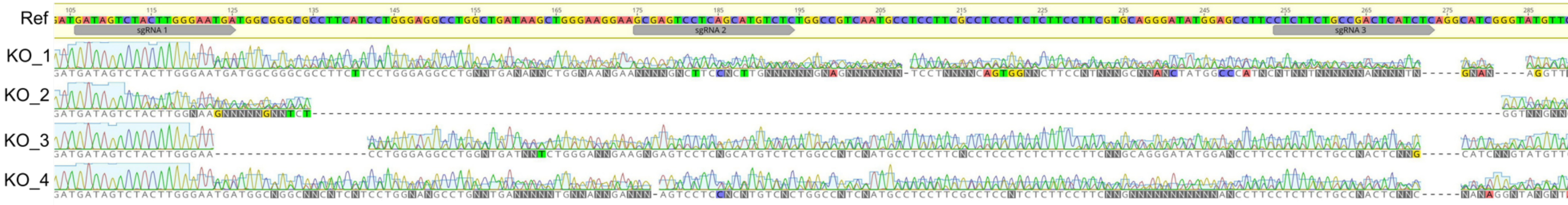

Fig S11: Genotyping of SV2B KO U937 Cells.
Sanger sequencing of locus targeted by gRNA in the CRISPR-induced U937 SV2B KO clones. Traces were aligned to WT reference sequence and heterogeneous mutations deconvoluted using ICE (https://ice.synthego.com/#/). (Left) KO scores as determined by ICE.
